# Global metabolic analyses identify key differences in metabolite levels between polymyxin-susceptible and polymyxin-resistant *Acinetobacter baumannii*

**DOI:** 10.1038/srep22287

**Published:** 2016-02-29

**Authors:** Mohd Hafidz Mahamad Maifiah, Soon-Ee Cheah, Matthew D. Johnson, Mei-Ling Han, John D. Boyce, Visanu Thamlikitkul, Alan Forrest, Keith S. Kaye, Paul Hertzog, Anthony W. Purcell, Jiangning Song, Tony Velkov, Darren J. Creek, Jian Li

**Affiliations:** 1 Drug Delivery, Disposition and Dynamics, Monash Institute of Pharmaceutical Sciences, Monash University, Parkville, VIC, 3052, Australia; 2 Department of Microbiology, Faculty of Medicine, Nursing & Health Sciences, Monash University, Clayton, VIC, 3800, Australia; 3 Faculty of Medicine Siriraj Hospital, Mahidol University, Bangkok, 10700, Thailand; 4 UNC Eshelman School of Pharmacy, The University of North Carolina at Chapel Hill, Chapel Hill, NC, 27599-7569, USA; 5 Detroit Medical Centre and Wayne State University, University Health Centre, Detroit, MI, 48201, USA; 6 Hudson Institute of Medical Research, Clayton, VIC, 3168, Australia; 7 Faculty of Medicine, Nursing & Health Sciences, Monash University, Clayton, VIC, 3800, Australia; 8 Department of Biochemistry and Molecular Biology, Faculty of Medicine, Nursing & Health Sciences, Monash University, Clayton, VIC, 3800, Australia

## Abstract

Multidrug-resistant *Acinetobacter baumannii* presents a global medical crisis and polymyxins are used as the last-line therapy. This study aimed to identify metabolic differences between polymyxin-susceptible and polymyxin-resistant *A. baumannii* using untargeted metabolomics. The metabolome of each *A. baumannii* strain was measured using liquid chromatography-mass spectrometry. Multivariate and univariate statistics and pathway analyses were employed to elucidate metabolic differences between the polymyxin-susceptible and -resistant *A. baumannii* strains. Significant differences were identified between the metabolic profiles of the polymyxin-susceptible and -resistant *A. baumannii* strains. The lipopolysaccharide (LPS) deficient, polymyxin-resistant 19606R showed perturbation in specific amino acid and carbohydrate metabolites, particularly pentose phosphate pathway (PPP) and tricarboxylic acid (TCA) cycle intermediates. Levels of nucleotides were lower in the LPS-deficient 19606R. Furthermore, 19606R exhibited a shift in its glycerophospholipid profile towards increased abundance of short-chain lipids compared to the parent polymyxin-susceptible ATCC 19606. In contrast, in a pair of clinical isolates 03–149.1 (polymyxin-susceptible) and 03–149.2 (polymyxin-resistant, due to modification of lipid A), minor metabolic differences were identified. Notably, peptidoglycan biosynthesis metabolites were significantly depleted in both of the aforementioned polymyxin-resistant strains. This is the first comparative untargeted metabolomics study to show substantial differences in the metabolic profiles of the polymyxin-susceptible and -resistant *A. baumannii*.

*Acinetobacter baumannii* is a Gram-negative, aerobic bacterium and a major cause of nosocomial infections worldwide, particularly in critically-ill patients^1^. *A. baumannii* infections include hospital-acquired pneumonia, bloodstream infection, urinary tract infection, skin and soft tissue infections^2^,^3^. *A. baumannii* has become a significant global threat and is one of the six ‘superbugs’ identified by the Infectious Diseases Society of America (IDSA) which required urgent attention for discovery of novel antibiotics^4^. Recently, the United States Centers for Disease Control and Prevention (CDC) classified multi-drug resistant (MDR) *A. baumannii* as a microorganism with a threat level of “Serious”^5^. *A. baumannii* has been characterised as ‘naturally transformable’, since it can rapidly acquire diverse resistance mechanisms and undergo genetic modifications that confer resistance to all current clinically used antibiotics^1^,^2^,^6^.

The clinical use of polymyxins waned in the 1970s due to potential nephrotoxicity and neurotoxicity^7^,^8^. However, over the last decade colistin (polymyxin E) and polymyxin B have been widely used as the only effective therapeutic option for patients infected with MDR *A. baumannii*^9^,^10^,^11^. Polymyxins are amphipathic, cationic lipopeptides that contain five L-α, γ-diaminobutyric acid (Dab) residues^7^,^8^. The bactericidal activity of polymyxins is exerted via the ‘self-promoted uptake’ pathway, initiated by electrostatic interaction with the lipid A of lipopolysaccharide (LPS) on the outer leaflet of the bacterial outer membrane^7^,^12^. In addition, a recent study suggested that polymyxins exert bacterial killing through a specific mechanism via the formation of hydroxyl radicals^13^. Polymyxin resistance in *A. baumannii* can be acquired via the addition of phosphoethanolamine^14^,^15^ or galactosamine^16^ to lipid A structure. Our group firstly reported that *A. baumannii* ATCC 19606 spontaneously acquired colistin resistance following exposure to high levels of colistin, via the loss of its initial target, LPS^17^. Further analyses revealed LPS loss was due to single random mutations in the lipid A biosynthesis genes, *lpxA, lpxC* and *lpxD*^17^. Moreover, transcriptomic analyses of the *A. baumannii* LPS-deficient strain 19606R revealed significant up-regulation of genes involved in the cell envelope and membrane biogenesis, in particular of the Lol lipoprotein transport system and the Mla-retrograde phospholipid transport system^18^. We therefore hypothesised that the LPS-deficient strain 19606R exhibits significant changes in its metabolic profile in response to LPS loss. For bacteria, metabolomics is a powerful systems biology tool for understanding cell physiology and can complement and validate data from genomics, transcriptomics and proteomics^19^,^20^,^21^. In this study, we report the first comparative untargeted metabolomics analyses of paired polymyxin-susceptible and polymyxin-resistant (via LPS loss or lipid A modifications) *A. baumannii* strains.

## Results

Comparative untargeted metabolomics was employed to identify differences in the metabolic profile between polymyxin-susceptible and polymyxin-resistant *A. baumannii* strains. Two pairs of *A. baumannii* strains were examined: a laboratory-derived polymyxin-resistant, LPS-deficient *lpxA*-mutant strain, 19606R and its polymyxin-susceptible parent strain, ATCC 19606; and two clinical isolates, polymyxin-susceptible 03–149.1 and polymyxin-resistant 03–149.2 obtained from a patient before and after colistin treatment, respectively. The polymyxin-resistant strain 19606R displayed a slower growth rate compared to the parent strain ATCC 19606, as previously reported^17^. Whereas, there was no significant difference in the growth rate between the paired polymyxin-susceptible 03–149.1 and polymyxin-resistant 03–149.2 clinical isolates.

### Genomics and lipid A structural analysis of *A. baumannii* clinical isolates 03–149.1 and 03–149.2

**Figure 1:**
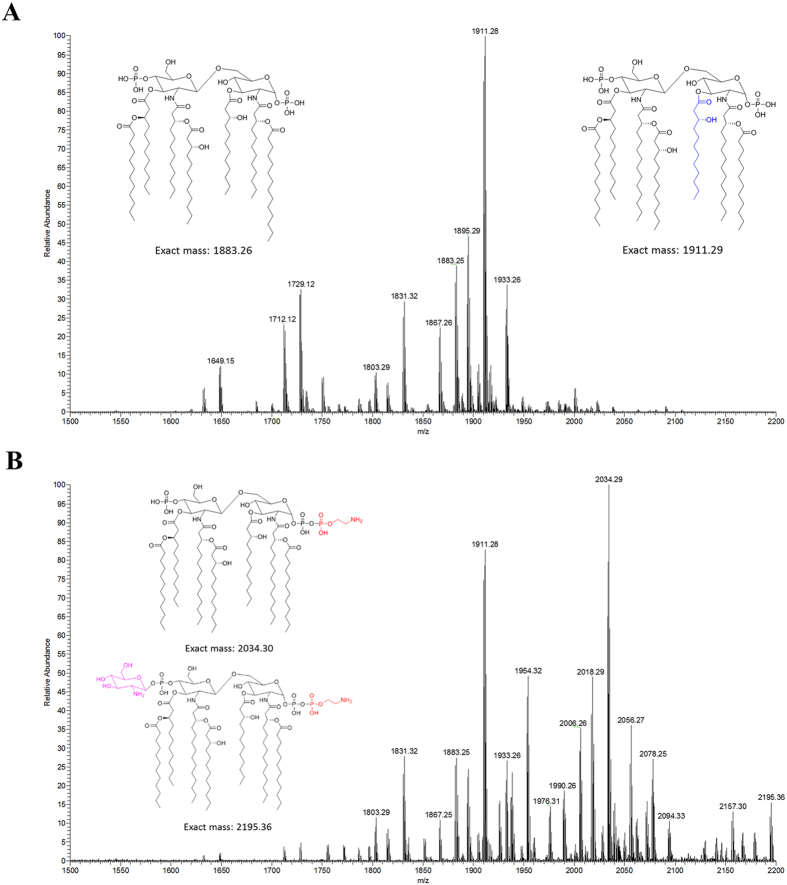
The mass spectra of lipid A isolated from the *A. baumannii* clinical isolates. (**A**) Polymyxin-susceptible 03–149.1 without lipid A modification. (**B**) Polymyxin-resistant 03–149.2 with lipid A modifications with phosphoethanolamine (pEtN) and galactosamine (GalN).

The paired *A. baumannii* clinical isolates of polymyxin-susceptible 03–149.1 and polymyxin-resistant 03–149.2 strains were initially identified using 16S rDNA gene sequencing; and showed 96.17% and 97.15% sequence similarity to the *A. baumannii* ATCC 19606, respectively (Supplementary Table S1). Furthermore, a comparison of the polymyxin-susceptible 03–149.1 and the polymyxin-resistant 03–149.2 by high-throughput sequencing and variant calling revealed 3 variations unique to the 03–149.2 isolate (Supplementary Table S2). One variation found in 03–149.2 was a deletion of 3 bases in the *pmrB* gene, which conferred an in-frame deletion of alanine 28. We also investigated the mechanism(s) of polymyxin resistance in the clinical isolate 03–149.2 with lipid A structural analysis. Lipid A samples isolated from both polymyxin-susceptible 03–149.1 and polymyxin-resistant 03–149.2 were characterised with electrospray ionization (ESI) high-resolution mass spectrometry in the negative-ion mode (Fig. 1). The mass spectrum of lipid A from the polymyxin-susceptible 03–149.1 shows a predominant peak at *m/z* 1911.28, which represents a hepta-acylated lipid A with four primary fatty acyls (i.e. two 3-hydroxylaurate [C_12_ (3-OH)] acyl chains and two 3-hydroxymyristate [C_14_ (3-OH)] acyl chains), and three secondary fatty acyls (i.e. one C_12_ (3-OH) acyl chain and two laurate (C_12_) acyl chains); while the peak at *m/z* 1933.26 represents the sodium adduct of the hepta-acylated lipid A mentioned above (Fig. 1A). The peak at *m/z* 1883.25 is for a hepta-acylated lipid A with four primary C_14_ (3-OH) acyl chains and three secondary fatty acyls (i.e. one C_12_ acyl chain, one C_12_ (3-OH) acyl chain, and one myristate (C_14_) acyl chain). The peak at *m/z* 1729.12 corresponds to a hexa-acylated lipid A, indicating the loss of a laurate acyl chain from the hepta-acylated lipid A at *m/z* 1911.28 (Δ*m/z* = −182). Additional peaks at *m/z* 1649.15, 1803.29, 1831.32 differ from the peaks listed above by dephosphorylation at the 1 or 4′ position of lipid A (Δ*m/z* = −80), while the peaks at *m/z* 1712.12, 1867.26, and 1895.29 were only different from the corresponding peaks at *m/z* 1729.12, 1883.25, and 1911.28 by the mass of one oxygen atom (Δ*m/z* = −16), indicating the absence of 3-hydroxylation at the secondary laurate acyl chain.

Mass spectrometry analyses of lipid A from the polymyxin-resistant 03–149.2 isolate revealed several different types of modifications in the lipid A structure (Fig. 1B). The predominant peak at *m/z* 2034.29 represents the hepta-acylated lipid A at *m/z* 1911.28 modified with a phosphoethanolamine (pEtN) residue (Δ*m/z* = +123), while the peak at *m/z* 1954.32 indicates its dephosphorylated form (Δ*m/z* = −80). Minor peaks at *m/z* 1990.26, 2006.26, and 2018.29 correspond to lipid A at *m/z* 1867.26, 1883.25, and 1895.29 which were modified with a pEtN group, respectively. The peak at *m/z* 2157.30 represents a modified lipid A with the addition of two pEtN moieties to the parent structure at *m/z* 1911.28. Interestingly, lipid A modified with galactosamine (GalN) was also detected in polymyxin-resistant 03–149.2. In detail, the peaks at *m/z* 2078.25 and 2094.33 represent lipid A at *m/z* 1895.29 and 1911.28 modified with a GalN residue (Δ*m/z* = +161) along with a sodium adduct, respectively, and the peak at *m/z* 2195.36 corresponds to a lipid A (*m/z* 1911.28) with both pEtN and GalN additions (Δ*m/z* = +284) (Fig. 1B).

### Optimal metabolite recovery of MDR *A. baumannii* by washing with 0.9% NaCl and extraction using chloroform:methanol:water (1:3:1)

**Figure 2:**
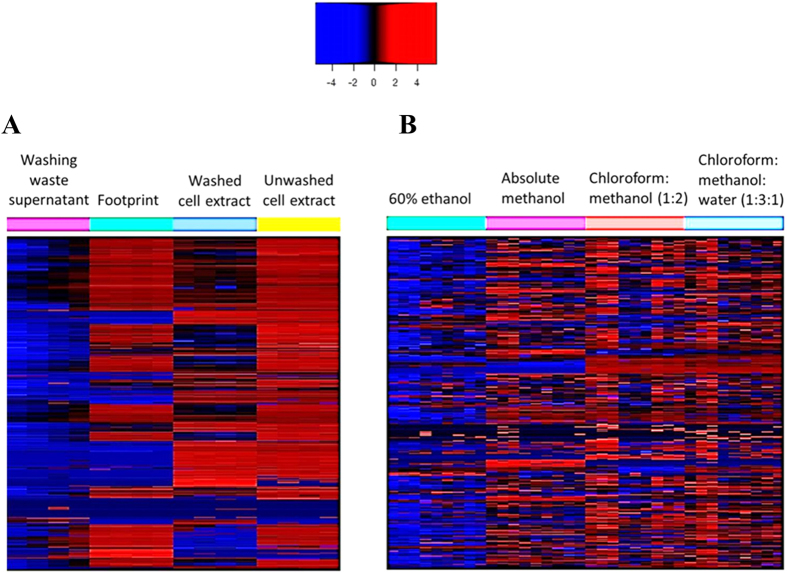
Evaluation of washing step and extraction solvents in the sample pre-treatment method. (**A**) Clustered heat map distinguished the total metabolite recovery between cells subjected to washing with 0.9% NaCl and without washing. (**B**) Comparison of four different extraction solvents on the global metabolite recovery in *A. baumannii*: 60% ethanol, absolute methanol, chloroform:methanol (1:2, v/v), and chloroform:methanol:water (1:3:1, v/v).

Optimisation of the metabolite sampling method was performed prior to the metabolomics analysis of paired polymyxin-susceptible and polymyxin-resistant strains. The potential for metabolite leakage during the washing step was examined by comparing washed and unwashed cell extracts, and analysing the washing waste supernatant. The hierarchical clustered heat map demonstrated that the washing step with 0.9% NaCl successfully removed the majority of culture media components (Fig. 2A). Recovery of intracellular metabolites (those not present in the broth) was not substantially impacted by washing. Analysis of the supernatant from the washing waste detected leakage of certain cell-derived metabolites, but at very low levels relative to the levels within the cell pellets. Furthermore, evaluation of four extraction solvents showed a total of 1099, 1104, 1070 and 1089 metabolites detected from the LC-MS analyses of metabolite samples extracted by the chloroform:methanol (CM; 1:2, v/v), chloroform:methanol:water (CMW; 1:3:1, v/v), 60% ethanol (60EtOH) and absolute methanol (MeOH) solvents, respectively. CMW was the most promising solvent, demonstrating efficient extraction of a wide range of metabolite classes (Fig. 2B). In addition, the median relative standard deviation (RSD) for all metabolites in the CMW samples was 22%, which is within an acceptable range and is comparable to the standard MeOH extraction solvent (Table 1). In comparison, CM and 60EtOH extraction solvents showed median RSD values of 25% and 24%, respectively. Peak intensities and RSD values for a number of common metabolites are provided in Table 1, showing that CMW was the most reproducible compared to the other three extraction solvents.

### Multivariate and univariate metabolomics analyses were able to identify key differences between the polymyxin-susceptible and polymyxin-resistant *A. baumannii* strains

**Figure 3:**
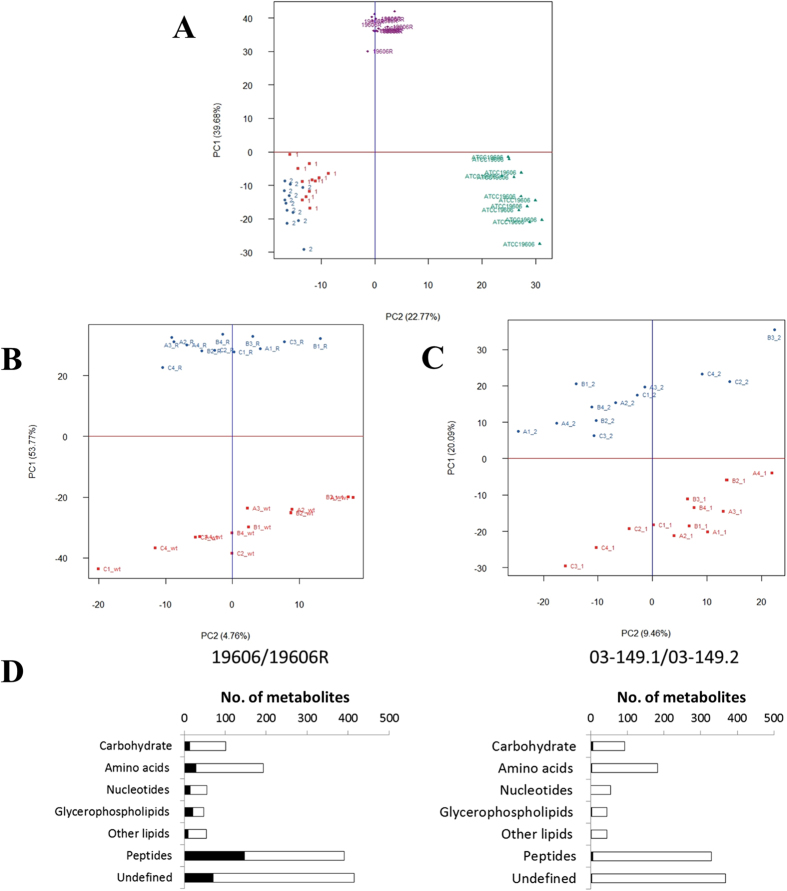
(**A**) PCA score plot of four *A. baumannii* strains. (**B**) PCA score plot of paired polymyxin-resistant 19606R and the wild-type ATCC 19606. (**C**) PCA score plot of paired polymyxin-resistant 03–149.2 and polymyxin-susceptible 03–149.1 clinical isolates. Each data set for individual strains represents a total of 12 sample replicates (3 biological replicates and each with 4 technical replicates). (**D**) Pathway-focused representation of the significant metabolites (black bars) and total number of putatively identified metabolites (open bars) for the polymyxin-resistant 19606R relative to the wild-type ATCC 19606 (left) and the polymyxin-resistant clinical isolate 03–149.2 relative to the polymyxin-susceptible isolate 03–149.1 (right). Significant metabolites were selected by at least 2-fold difference (*p* < 0.05) in the metabolites levels of polymyxin-resistant strain relative to the polymyxin-susceptible strain.

**Figure 4:**
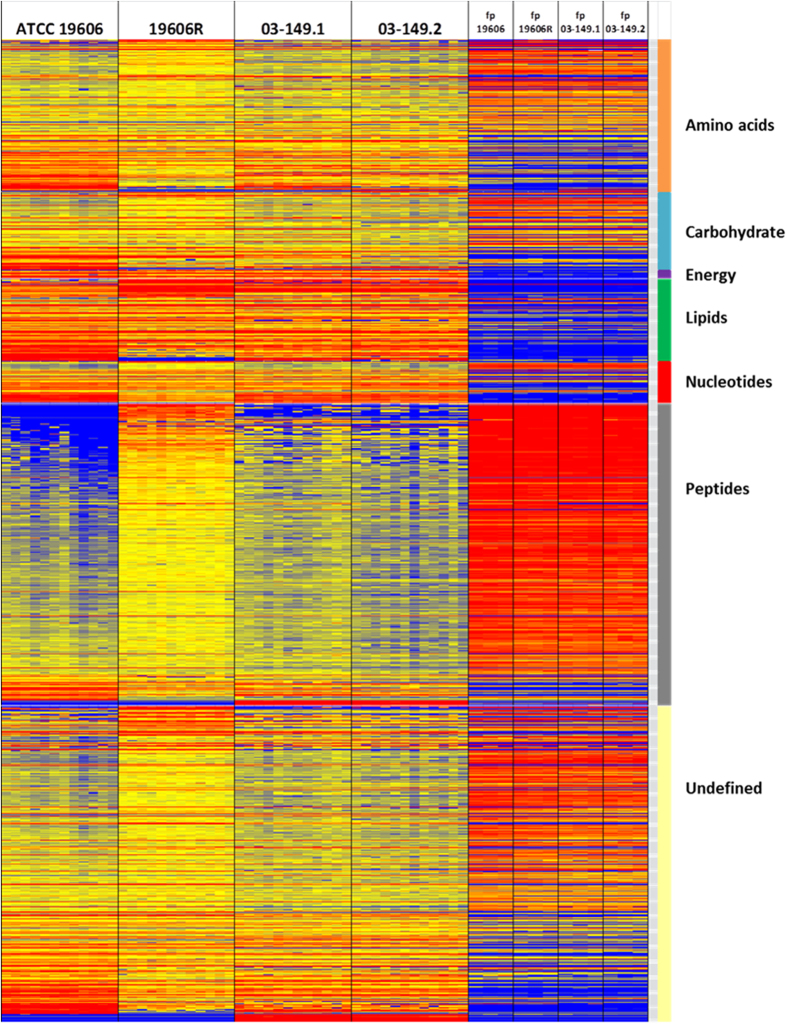
Heat map profiles of metabolite peak intensities in *A. baumannii*. Left: paired strains of ATCC 19606 and polymyxin-resistant 19606R, Right: paired clinical isolates polymyxin-susceptible 03–149.1 and polymyxin-resistant 03–149.2. Metabolites are grouped into different classes: amino acids, carbohydrates, energy, lipids, nucleotides, peptides and undefined. Metabolites derived from the footprint (fp) also represented in the heat map. The colors indicate the relative abundance of metabolites based on the relative peak intensity (red = high, yellow = no change, blue = undetectable).

The metabolomics data from the present LCMS-based comparative untargeted metabolomics study were highly reproducible. The pooled quality control samples clustered tightly in the PCA plot, indicating small analytical variations among the samples (Supplementary Figure S1). Furthermore, the median RSD value for all metabolites in this study was less than 10%. Global metabolome differences between four *A. baumannii* strains were visualised using PCA score plots (Fig. 3A) and heat map profiles (Fig. 4), and demonstrate that the polymyxin-resistant and polymyxin-susceptible strains differed significantly in their levels of a number of key cellular metabolites. PCA score plots also clearly show that there were global metabolic differences between the paired *A. baumannii* strains (Fig. 3B,C). Interestingly, nearly 25% of metabolites in the LPS-deficient polymyxin-resistant strain 19606R, were significantly more abundant than the corresponding polymyxin-susceptible parent strain ATCC 19606 (Fig. 3D). Peptides were highly enriched in 19606R, and it appears that many of the more abundant metabolites in this polymyxin-resistant strain 19606R were derived from the growth medium (Fig. 4). The accumulation of medium components within cells was unique to the LPS-deficient 19606R, and was not apparent in the polymyxin-resistant clinical isolate 03–149.2.

For univariate analyses, all the putatively identified cellular metabolites (i.e. those more abundant in cell pellets than in footprint samples) were further analysed to reveal those that showed at least 2-fold differences (*p < 0.05 and **p < 0.01) in relative abundance between the polymyxin-resistant and polymyxin-susceptible *A. baumannii* strains. Several cellular metabolites were differentially abundant in the polymyxin-susceptible ATCC 19606 and polymyxin-resistant 19606R strains including carbohydrate, amino acid, nucleotide and lipid metabolites. In comparison, there were very few metabolic differences observed in the polymyxin-resistant clinical isolate 03–149.2 and polymyxin-susceptible clinical isolate 03–149.1 (Fig. 3D).

### Perturbations in sugar and nucleotide metabolism

**Figure 5:**
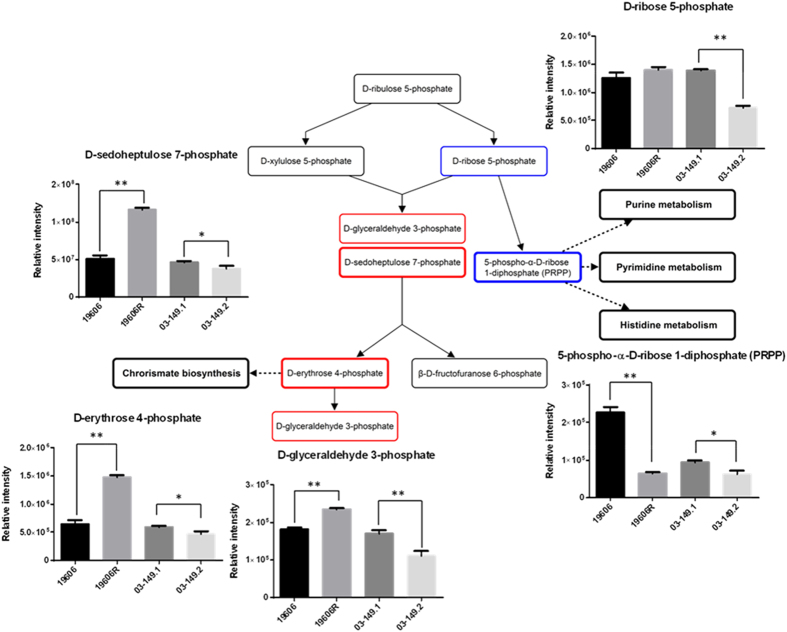
Pertubations of identified sugar phosphates in *A. baumannii*. Pentose phosphate pathway (PPP) of *A. baumannii*. PPP intermediates showed significant differences between polymyxin-resistant and polymyxin-susceptible *A. baumannii* strains. Metabolites in the red bold box indicate metabolites that were at least 2-fold more abundant in polymyxin-resistant 16906R strain than polymyxin-susceptible ATCC 19606 strain. Metabolites in the red box indicate metabolites that were less than 2-fold more abundant in the 19606R strain. The blue bold box indicates the metabolite that was at least 2-fold less abundant in 16906R than ATCC 19606. The blue box indicates the metabolite that was less than 2-fold less abundant in the 03–149.2 polymyxin-resistant strain than polymyxin-susceptible 03–149.1 strain. The black boxes indicate metabolites that were not detected. *p < 0.05; **p < 0.01.

**Figure 6:**
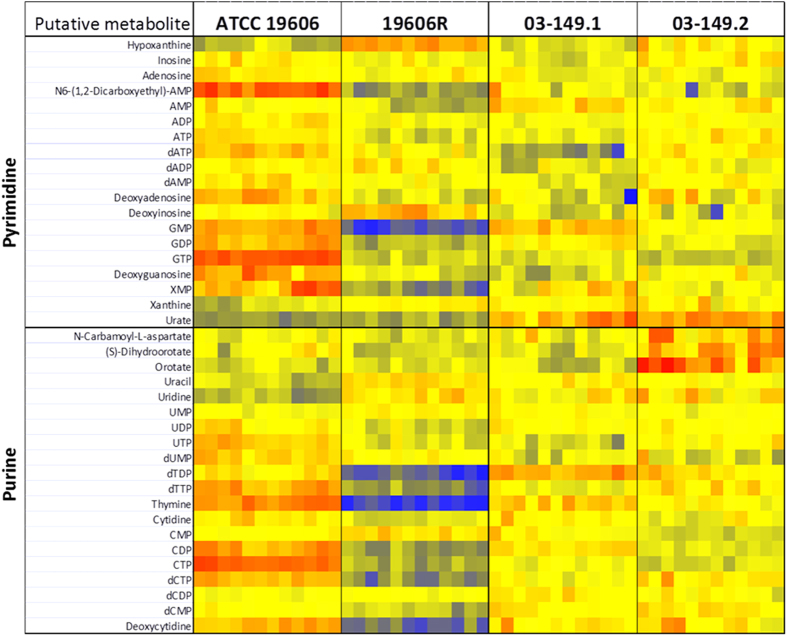
Heat map profiles of relative abundance of nucleotides. The polymyxin-resistant 19606R and its parent ATCC 19606 (left) and the clinical isolates polymyxin-resistant 03–149.2 and polymyxin-susceptible 03–149.1 (right). The colors indicate the relative abundance of metabolites based on the relative peak intensity (red = high, yellow = no change, blue = undetectable).

**Figure 7:**
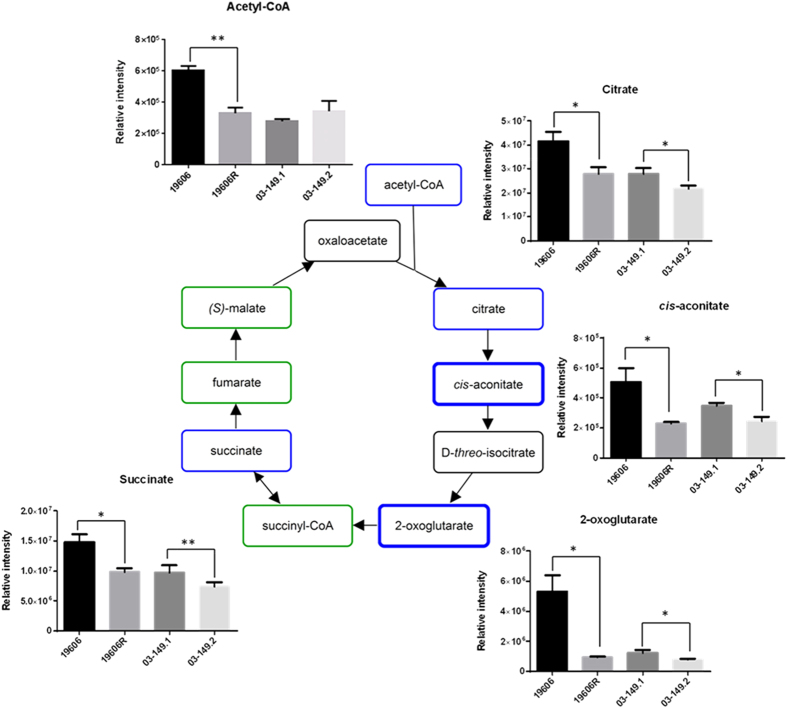
Pertubations of TCA cycle intermediates in *A. baumannii*. TCA cycle of *A. baumannii.* The blue bold box indicates metabolites that were at least 2-fold less abundant in the polymyxin-resistant 19606R strain than polymyxin-susceptible ATCC 19606. Metabolites in the blue box indicate metabolites that were less than 2-fold less abundant in 19606R than ATCC 19606. The green boxes indicate metabolites that were detected but not significant. The black boxes indicate metabolites that were not detected. *p < 0.05; **p < 0.01.

The polymyxin-resistant strain 19606R showed significant pertubations of several putative sugar phosphate metabolites, including metabolites associated with the pentose phosphate pathway (PPP). In particular, over 2-fold (*p* < 0.01) higher levels were observed for two PPP metabolites, D-erythrose 4-phosphate and D-sedoheptulose 7-phosphate, whereas the PPP-derived nucleotide precursor, 5-phospho-α-D-ribose 1-diphosphate (PRPP) was more than 3-fold (*p* < 0.01) lower than the polymyxin-susceptible parent strain ATCC 19606 (Fig. 5). On the contrary, the polymyxin-resistant clinical isolate 03–149.2 showed significantly lower abundance of the detected PPP metabolites, D-erythrose 4-phosphate, D-sedoheptulose 7-phosphate, D-glyceraldehyde 3-phosphate, and D-ribose 5-phosphate (*p* < 0.05) than the paired susceptible isolate 03–149.1. Besides, the levels of most nucleotides were significantly lower in the polymyxin-resistant 19606R than the parent polymyxin-susceptible ATCC 19606 strain (Fig. 6). However, there were no clear differences in nucleotide levels between the paired clinical isolates. Furthermore, two essential tricarboxylic acid (TCA) cycle intermediates, 2-oxoglutarate and *cis*-aconitate were identified at least 2-fold (*p* < 0.05) lower in relative abundance in the polymyxin-resistant 19606R cells (Fig. 7). Other TCA cycle metabolites, acetyl-CoA, citrate, and succinate, showed a consistent pattern of lower relative abundance in the polymyxin-resistant strain 19606R, albeit with less than two-fold difference. Interestingly, a similar pattern of metabolic changes were observed in the polymyxin-resistant clinical isolate 03–149.2; which showed lower abundance of citrate, *cis*-aconitate, 2-oxoglutarate and succinate than the polymyxin-susceptible clinical isolate 03–149.1.

### Variations of amino acid related metabolites in the polymyxin-resistant 19606R

**Figure 8:**
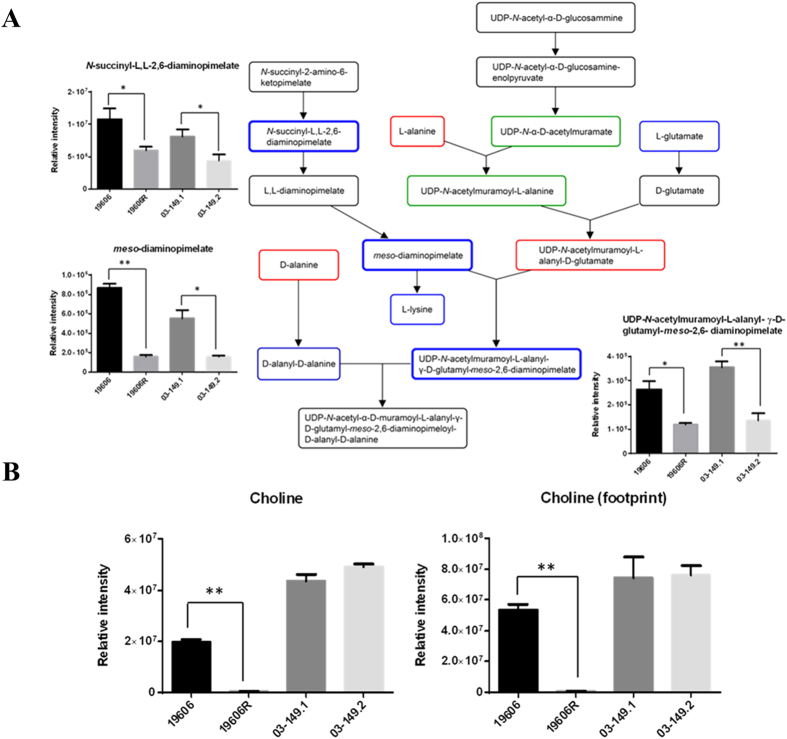
Levels of peptidoglycan biosynthesis metabolites and choline in *A. baumannii*. (**A**) Peptidoglycan synthesis pathway of *A. baumannii*. Metabolites in the red box indicate metabolites that were less than 2-fold higher in polymyxin-resistant strains than polymyxin-susceptible strains. The blue and bold boxes indicate metabolites that were at least 2-fold lower in polymyxin-resistant strains than polymyxin-susceptible strains. Metabolites in the blue box indicate less than 2-fold lower abundance in polymyxin-resistant strains than polymyxin-susceptible strains. The green box indicates metabolites that were detected but not significant. Metabolites in the black box were not detected. (**B**) Intracellular and footprint (extracellular) choline shows significantly lower abundance in the polymyxin-resistant 19606R than the parent wild-type ATCC 19606. *p < 0.05; **p < 0.01.

The abundance of several metabolites involved in phenylalanine, tyrosine, tryptophan and histidine metabolic pathways were significantly (*p* < 0.01) perturbed in the polymyxin-resistant 19606R strain (Table 2). Most notably, two putative metabolites associated with the shikimate pathway, shikimate-3-phosphate and 5-O-(1-Carboxyvinyl)-3-phosphoshikimate were significantly higher in abundance (between 11- to 14-fold) in the polymyxin-resistant 19606R compared to the polymyxin-susceptible parent strain ATCC 19606. Significant depletion was observed in three important peptidoglycan biosynthesis intermediates, *N*-succinyl-L,L-2,6-diaminopimelate, *meso*-diaminopimelate and UDP-*N*-acetylmuramoyl-L-alanyl-γ−D-glutamyl-*meso*-2,6-diaminopimelate which were 2- to 5-fold lower in the polymyxin-resistant 19606R (Fig. 8A). In addition, levels of these metabolites also decreased in the clinical polymyxin-resistant strain, 03–149.2. Interestingly, choline was undetectable in the polymyxin-resistant 19606R strain (Fig. 8B), suggesting differential uptake or utilisation of this metabolite from the growth medium. Footprint analysis revealed complete depletion of choline from the growth medium for the polymyxin-resitant 19606R, but not for the polymyxin-susceptible ATCC 19606 or both of the clinical isolates.

### Perturbation of lipids levels in the LPS-deficient polymyxin-resistant 19606R

**Figure 9:**
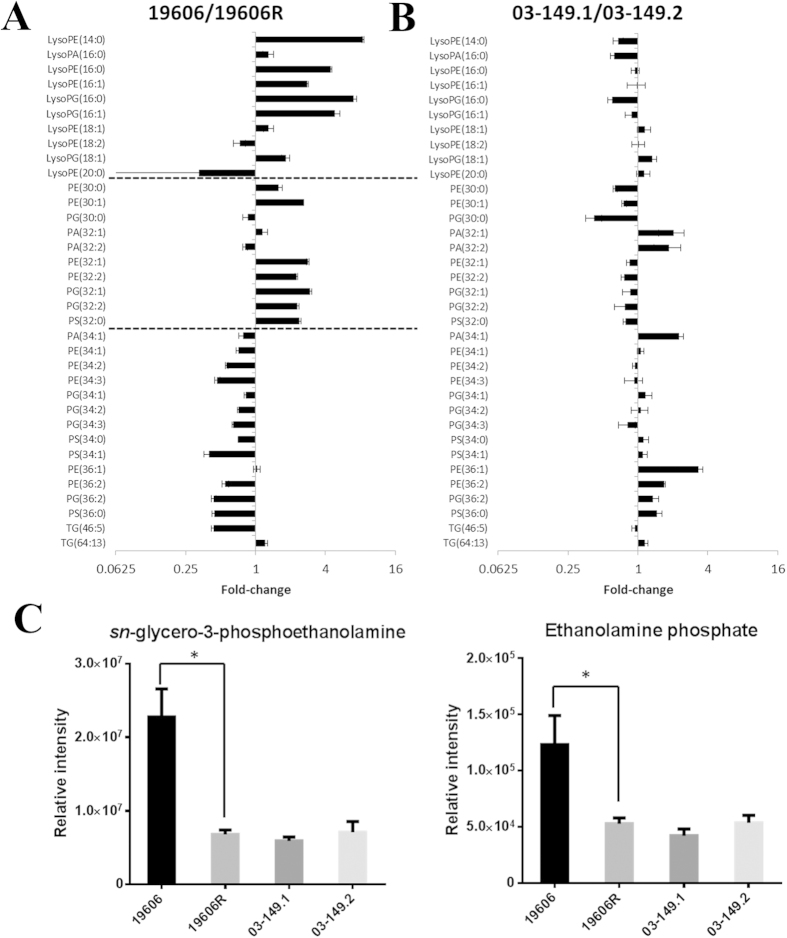
Relative intensity of glycerophospholipids levels. (**A**) LPS-deficient polymyxin-resistant 19606R and its parent strain ATCC 19606; and (**B**) polymyxin-resistant 03–149.2 and polymyxin-susceptible 03–149. (**C**) Glyceroethanolamine phosphate and ethanolamine phosphate showed significantly lower levels in the polymyxin-resistant 19606R than its parent ATCC 19606. PE, glycerophosphoethanolamine; PS, glycerophosphoserine; PG, glycerophosphoglycerols; PA, glycerophosphates. Fatty acyl carbon atom number and double bond number are shown in brackets. *p < 0.05.

Analyses of cellular lipid metabolites in the polymyxin-resistant 19606R and polymyxin-susceptible ATCC 19606 revealed profound alteration (*p* < 0.05) of several putatively identified lipid metabolites. The observed accurate masses and retention times indicated that many of these lipids were unsaturated and oxidised fatty acids; precise identification of these fatty acids is beyond the scope of this study. High level identification of glycerophospholipids (GPs) based on molecular formula revealed signficant perturbations in the major phospholipid species, glycerophosphoethanolamine (PE), glycerophosphoserine (PS), and glycerophosphoglycerol (PG). In general, GPs with shorter-chain fatty acids (total ≤ 32 carbons) were enriched in the polymyxin-resistant LPS-deficient 19606R, in addition to the shorter-chain lysophospholipids (≤18 carbons) (Fig. 9A). Notably, lipids with longer-chain fatty acids (>32 carbons) were generally depleted in the LPS-deficient 19606R. However, these trends were not observed in *A. baumannii* of both clinical isolates, polymyxin-susceptible 03–149.1 and polymyxin-resistant 03–149.2 (Fig. 9B). Furthermore, two key metabolites linked with glycerophospholipid metabolism, ethanolamine phosphate and glyceroethanolamine phosphate were significantly (*p* < 0.05) lower in abundance in the polymyxin-resistant 19606R than the polymyxin-susceptible parent strain ATCC 19606, but not significantly changed in both *A. baumannii* clinical isolates (Fig. 9C).

### Untargeted analysis reveals unknown metabolites that are common to both polymyxin-resistant strains

Four unidentified features were uniquely detected in both polymyxin-resistant strains and not in either of the polymyxin-susceptible strains. Whilst these features could not be identified based on existing bacterial metabolite databases, formula determination based on accurate mass, isotope abundance and retention time suggests that these unique metabolites may be complex amino-sugars: C_12_H_24_N_2_O_8_ (mass 324.153; t_R_ 14.7 min), C_30_H_57_N_2_O_12_P_3_ (mass 730.312; t_R_ 17.2 min), C_13_H_26_N_2_O_6_ (mass 306.179; t_R_ 19.8 min), and C_16_H_28_N_2_O_11_[Cl-] (mass 460.146; t_R_ 13.7 min). Notably, three unidentified metabolites were detected in both polymyxin-susceptible strains, but were absent in both of the polymyxin-resistant strains. Accurate mass indicates that these features likely represent metabolites with the formulas C_9_H_14_N_2_O_5_S (mass 262.063; t_R_ 13.4 min), C_7_H_11_NO_3_S (mass 189.046; t_R_ 7.5 min) and C_11_H_19_N_3_O_7_S_2_ (mass 369.066, t_R_ 16.8 min). The latter formula corresponds to γ-glutamyl cystine, and the presence of sulfur in the other formulas suggest that they may also be cysteine conjugates.

## Discussion

In recent times, untargeted metabolomics has been successfully applied towards the investigation of global metabolic profiles, particularly in microbiology and pharmacology^22^,^23^,^24^. Advantageously, the untargeted metabolomics platform enables the detection of both known and unknown metabolites and has allowed the elucidation of complex interactions between cellular metabolites^21^. Significantly, this global metabolomics approach has been beneficial in enhancing our understanding of the biological nature of antimicrobial resistance mechanisms. Global metabolic profiling distinguished differential metabolic patterns between antibiotic-susceptible and antibiotic-resistant strains of *A. baumannii, Pseudomonas aeruginosa, Nocardiopsis* spp., as well as the protozoan parasites *Trypanosoma brucei* and *Leishmania donovani*^25^,^26^,^27^,^28^,^29^. In a previous study, both planktonic and biofilm forms of *A. baumannii* were compared to identify metabolic profiles associated with biofilm synthesis^25^. In the present study, we employed a global metabolic profiling strategy to identify key metabolic differences between two pairs of polymyxin-susceptible and polymyxin-resistant *A. baumannii* strains, specifically conferred by two different mechanisms of polymyxin resistance, LPS loss and lipid A modifications.

Gram-negative bacteria can develop resistance to most current antimicrobial agents because of their extraordinary metabolic versatility and adaptability to a wide range of environmental conditions^2^. The main mechanisms used for conferring polymyxin resistance in Gram-negative bacteria involved modifications of lipid A, the membrane embedded component of lipopolysaccharides (LPS)^7^. In a previous study, we discovered that *A. baumannii* can develop resistance to very high colistin concentrations through a complete loss of LPS, due to spontaneous mutations in any one of the three key lipid A biosynthetic genes^17^. Polymyxin resistance in 19606R was shown to be conferred by a spontaneous single mutation in *lpxA* gene, resulting in LPS loss^17^. RNA expression profiling of polymyxin-resistant 19606R by our group indicated that significant outer-membrane remodelling occurs due to LPS loss^18^. This included increased expression of genes involved in cell envelope and membrane biogenesis, in particular the Lol lipoprotein transport system, the Mla-retrograde phospholipid transport system and poly-β-1,6-*N*-acetylglucosamine (PNAG) biosynthesis^18^. In addition, polymyxin-resistant 19606R displays a decreased expression of genes predicted to encode the fimbrial subunit FimA and components involved in the type VI secretion system (T6SS)^18^.

Our genome sequencing data for the polymyxin-resistant isolate 03–149.2 show that the deletion of 3 bases in the *pmrB* gene conferred an in-frame deletion of alanine 28. This particular mutation has not been characterized previously. However, mutations in *pmrB* have repeatedly been shown to cause polymyxin resistance in *A. baumannii* by the upregulation of the phosphoethanolamine transferase, *pmrC*, and subsequent lipid A modification^30^. Furthermore, structural analyses of lipid A from both polymyxin-susceptible 03–149.1 and polymyxin-resistant 03–149.2 clinical isolates revealed lipid A modifications with phosphoethanolamine (pEtN) and galactosamine (GalN) in the polymyxin-resistant 03–149.2 strain (Supplementary Figure S2). These lipid A modifications play a role in polymyxin resistance similar to that of aminoarabinose modification in other Gram-negative bacteria^31^,^32^ which reduce the initial electrostatic interaction with polymyxins by reducing the negative charge on the bacterial outer membrane^7^,^33^,^34^. The results clearly indicate that the mechanism of polymyxin resistance in the polymyxin-resistant 03–149.2 isolate differs from the *A. baumannii* 19606R resistant strain, which was due to the complete loss of LPS^17^.

Careful assessment of sample preparation methods is an important pre-requisite step to generate physiological metabolome data based on the differences in cell composition and culture condition^35^. In our study, the effect of the washing step and the efficiency of four different extraction solvents were firstly examined. Since a very rich culture medium, cation-adjusted Mueller-Hinton broth (MHB) was used in this study, a washing process was essential to avoid medium effects and to ensure that detected metabolites solely derive from cells^36^. Desirably, the leakage of intracellular metabolites into its extracellular environment should be avoided during the washing step^37^. As washing with organic solvents at sub-zero temperature leads to the leakage of cellular metabolites^38^, we implemented a quenching step at 0 °C and washing in aqueous buffer 0.9% NaCl (4 °C). The washing process was effective in eliminating most of the extracellular contaminants from the rich growth media whilst avoiding significant leakage of intracellular metabolites. Furthermore, the ideal extraction solvent should be able to extract a broad range of metabolites with different physicochemical properties in high and reproducible yield^39^. Several extraction solvents have been reported in the literature for bacterial metabolomics, and four promising solvent compositions were selected to determine the optimal extraction method specifically for *A. baumannii* in the present study. Unsurprisingly, our analyses showed that different extraction solvents preferentially extract certain metabolites depending on the polarity of the solvent. Overall, CMW (1:3:1, v/v) provided the greatest recovery and reproducibility for the largest number of different classes of metabolites, and is suitable to be used as a one-step method for untargeted metabolomics studies of *A. baumannii*.

Metabolic fingerprinting of two pairs of polymyxin-susceptible and polymyxin-resistant *A. baumannii* strains demonstrated accumulation or depletion of specific metabolite pools, indicating differential regulation of particular metabolic pathways. Interestingly, PCA plots clearly distinguished the metabolic profile differences between the polymyxin-resistant strain 19606R and the three other *A. baumannii* strains, signifying that the metabolic differences were substantially driven by the complete loss of outer membrane LPS. Notably, there were clear metabolic differences between the polymyxin-susceptible ATCC 19606 and polymyxin-resistant 19606R. In contrast, relatively very few metabolite differences were identified between the *A. baumannii* clinical isolates, polymyxin-susceptible 03–149.1 and polymyxin-resistant 03–149.2, demonstrating that lipid A modifications had minimal impact on the global metabolic profile. In general, the results show that different mechanisms of polymyxin resistance lead to unique changes in global metabolic profiles.

Our results demonstrate that peptides derived from the medium component were substantially accumulated in the polymyxin-resistant strain 19606R compared to other *A. baumannii* strains, and suggested that the uptake was facilitated significantly as a result of loss membrane integrity from the total LPS loss. The analyses of carbohydrate associated metabolites displayed higher levels of pentose phosphate pathway (PPP) intermediates in the polymyxin-resistant 19606R than in its susceptible parent strain ATCC 19606. In contrast, the polymyxin-resistant 03–149.2 showed significantly lower levels of detected PPP-associated metabolites than the polymyxin-susceptible 03–149.1. However, a major end-product of the PPP, 5-phospho-α-D-ribose 1-diphosphate (PRPP), was 3.5-fold lower in abundance in the polymyxin-resistant 19606R, suggesting diversion of flux through the non-oxidative branch of the PPP. PRPP is an essential precursor for both purine and pyrimidine nucleotide biosynthesis as well as for the biosynthesis of amino acids histidine and tryptophan^40^. Coincidently, decreased levels of nucleotides was observed in 19606R, as well as depletion of two histidine metabolites, *N*-formimino-L-glutamate (24-fold) and urocanate (3-fold), which may be secondary to the decreased concentration of PRPP. However, the significant depletion of nucleotide levels was not observed in the polymyxin-resistant 03–149.2 clinical isolate. The increased level of D-erythrose 4-phosphate (2-fold) generated in the PPP of the polymyxin-resistant 19606R strain appears to facilitate biosynthesis of the aromatic amino acids: phenylalanine, tyrosine, and tryptophan through the shikimate pathway, as shown by the significant accumulation of two intermediates, shikimate-3-phosphate (14-fold) and 5-*O*-(1-Carboxyvinyl)-3-phosphoshikimate (11-fold)^41^. Apart from the importance of PPP to conserve stable carbon equilibrium, to generate nucleotide and amino acid biosynthesis precursors and to supply reducing molecules for anabolism, PPP also has been found to be essential in the biosynthesis of LPS in Gram-negative bacteria^42^. SHI, an enzyme that has been characterised in *Escherichia coli, P. aeruginosa*^43^ and *Helicobacter pylori*^44^ converts sedoheptulose 7-phosphate into the LPS precursor, glycero-manno-heptose 7-phosphate^43^,^45^,^46^. Interestingly, we identified that the level of this particular metabolite, D-sedoheptulose 7-phosphate was about 2-fold higher (**p < 0.01) than the other three *A. baumannii* strains. We hypothesised that, as the polymyxin-resistant 19606R is characterised by the total LPS loss^17^, the metabolite, sedoheptulose 7-phosphate was significantly accumulated in the cells since it was not converted into the LPS precursor. The TCA cycle is another essential central metabolic pathway in bacterial cells, providing substrates for energy and biosynthetic reactions, including precursors for lipids and amino acids^47^. Notably, both polymyxin-resistant strains, 19606R and clinical isolate 03–149.2 showed lower abundance of TCA cycle metabolites than their respective polymyxin-susceptible strains. This suggested that, in general, the polymyxin-resistant strains produced less energy through TCA cycle indicating lower cellular metabolism than the polymyxin-susceptible strains and this was significantly observed particularly in the polymyxin-resistant strains, 19606R.

Three intracellular metabolites engaged in the peptidoglycan biosynthesis pathway, *meso*-diaminopimelate, UDP-*N*-acetylmuramoyl-L-alanyl-γ-D-glutamyl-*meso*-2-6-diaminopimelate, and *N*-succinyl-L,L-2,6-diaminopimelate were detected 2- to 5-fold less abundant in the polymyxin-resistant 19606R strain, compared to the parent strain ATCC 19606. Interestingly, these metabolites were also significantly decreased (2- to 3-fold) in the polymyxin-resistant clinical isolate 03–149.2. *Meso*-diaminopimelate is derived from lysine degradation and is conjugated with UDP-*N*-acetylmuramoyl-L-alanyl-D-glutamate (catalysed by MurE ligase) in the cytoplasm to form UDP-*N*-acetylmuramoyl-L-alanyl-γ-D-glutamyl-*meso*-2-6-diaminopimelate^48^. This is followed by the addition of dipeptide D-alanyl-D-alanine to form UDP-*N*-acetylmuramoyl-L-alanyl-D-glutamyl-6-carboxy-L-lysyl-D-alanyl-D-alanine (catalysed by MurF ligase). In *E. coli*, the MurE and MurF ligases are encoded by the *murE* and *murF* genes, respectively, co-localised in the genome; these ligases are essential for bacterial viability and are targets for antibacterial chemotherapy^49^. The lower levels of the peptidoglycan biosynthesis metabolites indicate that the polymyxin-resistant 19606R and clinical isolate 03–149.2 synthesised less peptidoglycan compared to their polymyxin-susceptible parent strains. Interestingly, choline levels were significantly depleted in the 19606R strain and its culture medium. In our recent transcriptomics study, the expression of choline dehydrogenase, choline-glycine betaine transporter and choline transport protein BetT was significantly increased (3.0, 3.0 and 4.6 folds, respectively) in the polymyxin-resistant *A. baumannii* 19606R^18^. As choline uptake and metabolism have been associated with maintenance of osmotic balance in Gram-negative bacteria^45^,^50^, our transcriptomics and metabolomics data collectively indicate that choline was required by 19606R in response to the osmolarity pressure due to the less peptidoglycan caused by polymyxin resistance. However, there was no profound change in choline level in the polymyxin-resistant clinical isolate 03–149.2 proposing that choline was not utilised and the level was in equilibrium state between intracellular and extracellular.

The outer membrane (OM) of Gram-negative bacterial cells is composed of an asymmetrical bilayer consisting of an outer leaflet with LPS as a major component, and the inner leaflet mainly containing glycerophospholipids (GPs)^51^. The OM serves as an efficient permeability barrier and a first-line defence mechanism, and GPs are the most prevalent component of lipids in the bacteria OM^52^. Compared to GPs species in the samples obtained from ATCC 19606, the LPS-deficient, polymyxin-resistant 19606R produced relatively high levels of GP species PE, PS and PG with shorter fatty acyl chains (less than 32 carbons in both chains) and concomitantly less GP species with more than 34 carbons in their fatty acyl chains. This finding agrees with a previous report that showed a LPS-deficient *Neisseria meningitides* mutant preferentially incorporated saturated PE and PG species with shorter fatty acyl chains into its OM^53^. Furthermore, the higher abundance of lyso-GPs (those with a single fatty acid chain less than 18 carbons) in the polymyxin-resistant 19606R, compared to the parent strain ATCC 19606, indicate significant GPs turnover; hence, our result supports the hypothesis that the OM structure of polymyxin-resistant bacterial cells is dramatically altered due to LPS loss. The observed increase in the production of GPs, which we hypothesise are mainly exported to the outer leaflet of the OM of the LPS-deficient strain 19606R, further supports the previously described the compensatory mechanism for the LPS loss which associated with increase in cell envelope and membrane biosynthesis^18^. Transcriptomics analyses of the LPS-deficient strain 19606R revealed that there was a significant increase in the expression of genes involved in phospholipid transport (*mlaBCD*) in response to the LPS loss^18^. Remarkably, glyceroethanolamine phosphate and ethanolamine phosphate showed significantly lower levels in the polymyxin-resistant 19606R than its parent ATCC 19606. Ethanolamine utilisation was suggested to associate with bacterial pathogenesis and virulence^54^,^55^. Our results support the claim and suggest that ethanolamine is crucial for bacterial metabolism, in particular in the polymyxin-resistant 19606R. The present study utilising HILIC chromatography does not represent the total phospholipid composition and does not reveal the relative distribution of each GP species in the inner and outer membranes of *A. baumannii*. Future membrane lipidomics analysis of LPS-deficient, polymyxin-resistant *A. baumannii* is underway and will further define the total lipid abundance and distribution.

In addition to the perturbations to known metabolic pathways, our untargeted metabolomics analysis revealed four unidentified metabolite features which are consistent with amino-sugars that were unique to the polymyxin-resistant strains, and not in either of the polymyxin-susceptible strains. Metabolite identification is a major bottleneck in untargeted metabolomics, and accurate identification of metabolites that are not present in existing databases requires large-scale fractionation and extensive structural analysis^56^,^57^,^58^. Precise structural identification of the unknown metabolites that are unique to polymyxin-resistant strains is beyond the scope of the present study. Nevertheless, with the high-resolution MS applied here, features can be annotated with the most likely molecular formulas. Whilst not conclusive, these unique unidentified metabolites suggest the involvement of glycan metabolism in the molecular mechanisms of polymyxin resistance in *A. baumannii*. Further studies are warranted to characterise these unknown metabolites and their biological functions. Together with our metabolomics and transcriptomics results^18^, it will provide additional information about the metabolic differences between polymyxin-susceptible and polymyxin-resistant *A. baumannii* (Figs 3 and 4).

To the best of our knowledge, this comparative untargeted metabolomics study is the first to demonstrate significant global metabolic changes in polymyxin-resistant *A. baumannii* strains. In particular, global metabolic differences are associated with different mechanisms of polymyxin resistance due to LPS loss and lipid A modifications. Our study provides a valuable insight into the global metabolism of polymyxin-resistant *A. baumannii* and potentially offers new therapeutic targets.

## Materials and Methods

### Strains

The *A. baumannii* wild-type strain ATCC 19606 was obtained from the American Type Culture Collection. The *lpxA* mutant strain 19606R (MIC > 128 mg/L) is an LPS-deficient, polymyxin-resistant derivative of ATCC 19606^17^. The two clinical isolates used in this study were polymyxin-susceptible 03–149.1 (MIC 1 mg/L) and polymyxin-resistant 03–149.2 (MIC > 32 mg/L); both were isolated from the same patient^59^. Bacterial strains were grown in cation-adjusted Mueller-Hinton broth (MHB; Oxoid, England; 20–25 mg/L Ca^2+^ and 10–12.5 mg/L Mg^2+^).

### Identification of 16S rDNA, genome sequencing and lipid A structural analysis of *A. baumannii* clinical isolates 03–149.1 and 03–149.2

The *A. baumannii* clinical isolates 03–149.1 and 03–149.2 16S were identified using rDNA gene sequencing (Supplementary Method). Their genome sequences were determined using 36-bp paired-end sequencing chemistry on an Illumina Genome Analyzer II apparatuse (Illumina) at the Micromon Sequencing Facility (Monash University) as previously described^17^. Furthermore, lipid A of the clinical isolates 03–149.1 and 03–149.2 was prepared by mild acid hydrolysis as previously described^60^. In detail, 100 mL of broth cultures were harvested at OD_600nm_ = 0.8 via centrifugation at 3,220 × *g* for 20 min and washed twice with phosphate-buffered saline (PBS). Initially, the cells were re-suspended in 4 mL PBS, methanol (10 mL) and chloroform (5 mL) were then added to the suspension, making a single-phase Bligh-Dyer (chloroform/methanol/water, 1:2:0.8, v/v)^61^. The mixture was centrifuged at 3,220 × *g* for 15 min and supernatant was removed. The pellet was washed once with chloroform/methanol/water (1:2:0.8, v/v), re-suspended in the hydrolysis buffer (50 mM sodium acetate pH 4.5, 1% sodium dodecyl sulphate (SDS)), and incubated in a boiling water bath for 45 min. To extract lipid A, the SDS solution was converted into a double-phase Bligh-Dyer mixture by adding 6 mL of chloroform and 6 mL of methanol for a final mixture of chloroform/methanol/water (1:1:0.9, v/v)^61^. The lower phase containing lipid A was finally extracted and samples were dried and stored at –20 °C. Structural analysis of lipid A was performed in negative mode on a Q-Exactive Hybrid Quadrupole-Orbitrap Mass Spectrometer (Thermo Fisher).

### Bacterial culture preparation for metabolomics experiments

Bacterial strains, subcultured from –80 °C frozen stocks, were inoculated onto nutrient agars and incubated for 16–18 h at 37 °C. For the polymyxin-resistant strains, *lpxA* mutant 19606R and clinical isolate 03–149.2, the Mueller-Hinton plates were supplemented with polymyxin B (10 mg/L) to maintain the selection pressure. For each culture, a single colony was used to inoculate 10 mL MHB for incubation overnight (16–18 h) at 37 °C with constant shaking (180 rpm). Three biological replicate reservoirs for different *A. baumannii* colonies, each consisting of 50 mL MHB, were prepared for each *A. baumannii* strain. Each reservoir was inoculated with 500 μL of overnight culture and grown at 37 °C with shaking (180 rpm) to an OD_600nm_ ~0.5 (mid-exponential growth phase). The polymyxin-resistant strains 19606R and 03–149.2 were grown in MHB without colistin. For the blank controls, two MHB reservoirs without bacterial inoculation were included in the experiment.

### Sample preparation for metabolomic study

Metabolomic sample was prepared as previously described with slight modifications^36^. The sample pre-treatment method, washing step and extraction solvents were optimised for improved recovery of cellular metabolites. The final method for cell pellet analyses employed four technical replicates, each consisting of 10 mL mid-exponential culture (OD_600nm_ ~0.5) collected in 50 mL Falcon tubes (Thermo Fisher). The tubes were centrifuged at 3,220 × *g* at 4 °C for 5 min and the supernatant discarded. For each sample, extracellular metabolites and medium components were removed by washing cell pellets twice with 0.5 mL of 0.9% NaCl (4 °C). Following each wash, cells were pelleted by centrifugation at 3,220 × *g* at 4 °C for 3 min. To evaluate the washing effect on the metabolite leakage, washing waste supernatant samples were collected and analysed (below). Furthermore, the efficiency of four different extraction solvents were evaluated: (i) absolute methanol (MeOH), (ii) 60% ethanol (60EtOH), (iii) chloroform:methanol:water (CMW; 1:3:1, v/v), and (iv) chloroform:methanol (CM; 1:2, v/v). In our comparison study of different *A. baumannii* strains, washed cell pellets were resuspended in 0.5 mL metabolite extraction solvent consisting of CMW (1:3:1, v/v; −80 °C); the solvent mixture contained the internal standards (CHAPS, CAPS, PIPES and TRIS; 1 μM of each). These compounds were selected as the internal standards as they are physicochemically diverse small molecules that are not naturally occurring in any microorganism and can be spiked at known concentrations to determine the analytical performance of the method used. Samples were frozen in liquid nitrogen and thawed on ice, and freeze-thaw was repeated three times in order to permeabilise the cells and release intracellular metabolites. The mixtures were centrifuged for 10 min at 3,220 × *g* at 4 °C and 300 μL of the supernatants containing the extracted metabolites were collected in 1.5-mL centrifuge tubes and stored at –80 °C immediately. For analysis, the samples were thawed and further centrifuged at 14,000 × *g* for 10 min at 4 °C and 200 μL of particle-free supernatant was transferred into the injection vial for LC-MS analysis. For footprint samples, an aliquot of approximately 1.5 mL of the culture was rapidly filtered through a 0.22-μm filter and stored at –80 °C. Prior to analysis, these samples were thawed and 10 μL combined with 250 μL extraction solvent (chloroform:methanol:water, 1:3:1, v/v) and then centrifuged at 14,000 × *g* for 10 min at 4 °C to collect 200 μL supernatant for LC-MS analysis (below). Equal volumes from each of the *A. baumannii* strains samples were mixed for a quality control sample (QC). This pooled quality control sample was used to estimate a composite sample profile representing all the analytes that will be encountered during the LC-MS analysis^62^.

### LC-MS analysis

Hydrophilic interaction liquid chromatography (HILIC)—high-resolution mass spectrometry (HRMS) was employed in this study. Samples were analysed on a Dionex high-performance liquid chromatography (HPLC) system (RSLCU3000, Thermo Fisher) using a ZIC-pHILIC column (5 μm, polymeric, 150 × 4.6 mm; SeQuant, Merck) coupled to a Q-Exactive Orbitrap mass spectrometer (Thermo Fisher) operated at 35000 resolution in both positive and negative electro-spray ionization (ESI) mode and a detection range of 85 to 1,275 *m/z*. The LC solvent consisted of 20 mM ammonium carbonate (A) and acetonitrile (B) with a multi-step gradient system from 80% B to 50% B over 15 min, then to 5% B at 18 min, followed by wash with 5% B for 3 min, and 8 min re-equilibration with 80% B at a flow rate of 0.3 mL/min^63^. The run time was 32 min and the injection sample volume was 10 μL. All samples (3 biological replicates, each with 4 technical replicates) were randomized and analysed in a single LC-MS batch to reduce batch-to-batch variation. The chromatographic peaks, signal reproducibility and analyte stability were monitored by assessment of pooled quality control sample analysed periodically throughout the run, internal standards and total ion chromatograms for each sample. Mixtures of pure standards containing over 250 metabolites of different classes were analysed within the batch to aid in the identification of metabolites.

### Data processing, bioinformatics and statistical analyses

Global metabolomics analyses were performed using mzMatch^64^ and IDEOM (http:// mzmatch.sourceforge.net/ideom.php) free software^65^. Raw LC-MS data were converted to mzXML format and chromatogram peaks were detected using XCMS^66^ and saved in the peakML format. The program Mzmatch.R was used to align samples and filter peaks based on minimum detectable intensity (100000), reproducibility (relative standard deviation (RSD) for all replicates < 0.5) and peak shape (codadw > 0.8). Mzmatch.R was also used to retrieve LC-MS peak intensities for missing peaks and for the annotation of related peaks. Unwanted noise and artefact peaks were eliminated using IDEOM with default parameters. Metabolites were putatively identified by the exact mass within 2 ppm, after correction for loss or gain of a proton in negative and positive ESI mode, respectively. Retention time was employed to confirm the identification of each metabolite based on the available authentic standards. Putative identification of other metabolites was determined using exact mass and predicted retention time based on the Kyoto Encyclopedia of Genes and Genomes (KEGG), MetaCyc and LIPIDMAPS databases, with preference given to bacterial metabolites annotated in EcoCyc. Quantification of each metabolite was calculated using the raw peak height and is expressed relative to the average peak height for their paired susceptible strain. Univariate statistical analyses utilised a Welch’s T-test (α = 0.01) and multivariate analyses utilised the metabolomics R package. Metabolic pathway analyses were performed using the free web-based metabolomics tool Pathos (http://motif.gla.ac.uk/Pathos/)^67^, BioCyc (http://biocyc.org/)^68^, and Visualization and Analysis of Networks containing Experimental Data (Vanted) software^69^.

## Additional Information

**How to cite this article**: Maifiah, M. H. M. *et al*. Global metabolic analyses identify key differences in metabolite levels between polymyxin-susceptible and polymyxin-resistant *Acinetobacter baumannii. Sci. Rep.* **6**, 22287; doi: 10.1038/srep22287 (2016).

## Supplementary Material

Supplementary Information

## Figures and Tables

**Table 1:** Validation and reproducibility of metabolite extraction procedure for four different extraction solvents.

	CM	CMW	60EtOH	MeOH
Median RSD value (%)	25	22	24	20
Identified compound				
Amino acids				
L-Lysine	4.1 × 10 ^7^ (20.7)	5.7 × 10^7^ (16.1)	4.1 × 10^7^ (20.4)	4.2 × 10^7^ (27.6)
L-Serine	1.7 × 10^7^ (22.2)	1.7 × 10^7^ (13.7)	1.3 × 10^7^ (15.7)	1.8 × 10^7^ (7.3)
L-Tyrosine	9.7 × 10^7^ (14.0)	9.5 × 10^7^ (12.8)	5.6 × 10^7^ (17.6)	1.1 × 10^8^ (11.2)
L-Methionine	1.8 × 10^8^ (11.9)	1.7 × 10^8^ (13.9)	1.2 × 10^8^ (14.5)	1.9 × 10^8^ (10.4)
L-Phenylalanine	3.0 × 10^8^ (13.4)	2.7 × 10^8^ (13.0)	1.8 × 10^8^ (10.8)	2.7 × 10^8^ (9.5)
L-Valine	1.0 × 10^7^ (14.1)	9.6 × 10^6^ (10.2)	7.4 × 10^6^ (13.7)	1.0 × 10^7^ (5.6)
L-Aspartate	2.8 × 10^7^ (11.8)	2.5 × 10^7^ (9.4)	2.2 × 10^7^ (10.5)	2.6 × 10^7^ (6.3)
Energy				
NADPH	5.4 × 10^5^ (45.0)	1.6 × 10^6^ (11.5)	3.8 × 10^5^ (48.3)	6.9 × 10^5^ (35.1)
NAD^+^	4.6 × 10^7^ (33.0)	7.6 × 10^7^ (9.8)	3.4 × 10^7^ (16.1)	5.5 × 10^7^ (23.4)
FMN	4.7 × 10^5^ (31.3)	6.2 × 10^5^ (7.6)	NA*	4.0 × 10^5^ (61.7)
Nucleotides				
Adenine	6.7 × 10^6^ (46.7)	5.1 × 10^6^ (15.6)	2.3 × 10^6^ (41.8)	8.8 × 10^6^ (26.6)
Cytidine	9.1 × 10^6^ (24.1)	8.7 × 10^6^ (11.9)	4.0 × 10^6^ (10.9)	8.1 × 10^6^ (10.1)
Guanine	2.1 × 10^5^ (38.6)	2.1 × 10^5^ (12.6)	1.5 × 10^5^ (28.2)	2.5 × 10^5^ (12.9)
Uridine	8.7 × 10^6^ (19.2)	8.2 × 10^6^ (14.0)	5.0 × 10^6^ (15.2)	1.0 × 10^7^ (17.3)
Carbohydrate				
Pyruvate	3.0 × 10^5^ (19.8)	3.0 × 10^5^ (17.0)	2.4 × 10^5^ (25.2)	2.8 × 10^5^ (10.7)
Sucrose	2.0 × 10^6^ (39.2)	1.9 × 10^6^ (13.5)	1.1 × 10^6^ (13.7)	1.8 × 10^6^ (14.7)
Citrate	1.7 × 10^7^ (38.7)	2.8 × 10^7^ (14.9)	2.7 × 10^7^ (20.4)	2.2 × 10^7^ (18.6)
*cis*-Aconitate	2.3 × 10^5^ (29.3)	4.5 × 10^5^ (14.9)	2.6 × 10^5^ (17.8)	3.3 × 10^5^ (19.5)
Oxalate	2.5 × 10^5^ (22.7)	2.7 × 10^5^ (19.3)	2.8 × 10^5^ (29.2)	2.3 × 10^5^ (22.9)
(R,R)-Tartaric acid	3.2 × 10^4^ (19.4)	4.0 × 10^4^ (17.3)	3.4 × 10^4^ (23.3)	3.3 × 10^4^ (22.3)

CM, chloroform:methanol (1:2, v/v); CMW, chloroform:methanol:water (1:3:1, v/v); 60EtOH, 60% ethanol; MeOH, absolute methanol (3 biological samples with 3 technical replicates per condition). *NA, not available as the metabolite was not detected. Data are expressed as mean relative intensity (relative standard deviation, RSD, %).

**Table 2:** Fold changes (relative intensity) in the abundance of metabolites detected in the LPS-deficient polymyxin-resistant 19606R, relative to the parent strain ATCC 19606.

Formula	Putative metabolite^a^	Pathway/metabolism	Fold change	*P-*value
Carbohydrate				
C_5_H_12_O_5_	Xylitol	Pentose and glucoronate interconversions	3.47	0.00017
C_12_H_23_O_14_P	Lactose 6-phosphate	Galactose metabolism	2.14	0.0018
C_3_H_6_O_9_P_2_	Cyclic 2,3-bisphospho-D-glycerate	Carbohydrate metabolism	–3.01	0.0035
Amino acids				
C_7_H_11_O_8_P	Shikimate 3-phosphate	Phenylalanine, tyrosine, tryptophan biosynthesis	14.41	0.0012
C_10_H_13_O_10_P	5-O-(1-Carboxyvinyl)-3-phosphoshikimate	Phenylalanine, tyrosine, tryptophan biosynthesis	11.30	0.0079
C_8_H_8_O_5_	3,4-Dihydroxymandelate	Tyrosine	3.03	0.00074
C_4_H_6_O_3_	2-Methyl-3-oxopropanoate	Valine, leucine and isoleucine degradation	2.19	0.00060
C_2_H_5_O_5_P	Acetyl phosphate	Taurine and hypotaurine	2.15	9.2E-05
C_9_H_8_O_3_	Phenylpyruvate	Phenylalanine	–2.00	5.9E-05
C_9_H_10_O_4_	3-(2,3-Dihydroxyphenyl)propanoate	Phenylalanine	–2.03	0.0011
C_13_H_15_NO_6_	4-Hydroxyphenylacetylglutamic acid	Tyrosine	–2.08	0.0077
C_7_H_15_NO_3_	L-Carnitine	Lysine degradation	–2.57	0.00049
C_6_H_6_N_2_O_2_	Urocanate	Histidine	–3.75	0.0031
C_6_H_10_N_2_O_4_	*N*-Formimino-L-glutamate	Histidine	–24.86	0.00095

^a^Putative metabolites, identified by exact mass, with at least 2-fold differences at *p* value < 0.01 between the polymyxin-resistant 19606R and the polymyxin-susceptible ATCC 19606.
