# Supplementary material for: Global metabolic analyses identify key differences in metabolite levels between polymyxin-susceptible and polymyxin-resistant Acinetobacter baumannii
Source: Sci Rep. 2016 Feb 29;6:22287. doi: 10.1038/srep22287 (PMC4770286; doi:10.1038/srep22287)
Supplement: Supplementary Information [file srep22287-s1.pdf]

Global metabolic analyses identify key differences in metabolite levels between polymyxin-susceptible and polymyxin-resistant *Acinetobacter baumannii*

Mohd Hafidz Mahamad Maifiah[1], Soon-Ee Cheah[1], Matthew D. Johnson[1], Meiling Han[1], John D. Boyce[2], Visanu Thamlikitkul[3], Alan Forrest[4], Keith S. Kaye[5], Paul Hertzog[6,7], Anthony W. Purcell[8], Jiangning Song[8], Tony Velkov[1], Darren J. Creek[1,†], Jian Li[1,†,*]

[1]Drug Delivery, Disposition and Dynamics, Monash Institute of Pharmaceutical Sciences, Monash University, Parkville, VIC, 3052, Australia.

[2]Department of Microbiology, Faculty of Medicine, Nursing & Health Sciences, Monash University, Clayton, VIC, 3800, Australia.

[3]Faculty of Medicine Siriraj Hospital, Mahidol University, Bangkok, 73170, Thailand.

[4]UNC Eshelman School of Pharmacy, The University of North Carolina at Chapel Hill, Chapel Hill, NC, 27599-7569, USA.

[5]Detroit Medical Centre and Wayne State University, University Health Centre, Detroit, MI, 48201, USA.

[6]Hudson Institute of Medical Research, Clayton, VIC, 3168, Australia.

[7]Faculty of Medicine, Nursing & Health Sciences, Monash University, Clayton, VIC, 3800, Australia.

[8]Department of Biochemistry and Molecular Biology, Faculty of Medicine, Nursing & Health Sciences, Monash University, Clayton, VIC, 3800, Australia.

†Joint senior authors: Jian Li, email: Colistin.Polymyxin@gmail.com, phone: +61 3 9903 9702, fax: +61 3 9903 9586; Darren J. Creek, email: darren.creek@monash.edu, phone: +61 3 9903 9249, fax: +61 3 9903 9583

*Corresponding author: Jian Li, email: Colistin.Polymyxin@gmail.com

Supplementary Information

Method for 16S rDNA gene sequencing

Degenerate oligonucleotide primers 16s_Fw CCTACGGGNGGCWGCAG and 16s_Rv GACTACHVGGGTATCTAATCC were used to amplify the 16S gene from each strain by PCR. The 16S fragment was purified by gel extraction (Qiagen) and sequenced using Sanger chemistry (Micromon, Monash University, Australia). Sequences were analysed using Eztaxon[1] and identification of phylogenetic neighbours was conducted using BLASTN[2] program against the database containing published prokaryotic representatives[1].

Supplementary Table S1. Phylogenetic characterisation by 16S gene sequencing

Strains name	Length (base pairs)[a]	Closest match[b]	Similarity %[c]	Completeness %[d]
03-149.1	446	ATCC 19606	96.17	30.6
03-149.2	442	ATCC 19606	97.15	30.4

[a] Length of input sequence generated by sanger-based sequencing methodologies.
[b] Closest match determined by BLASTn against a database of published prokaryotic representatives.
[c] Similarity based on BLASTn search against the closest match.
[d] Completeness accounts for the entire 16S gene of the closest match.

Supplementary Table S2. Mutations identified in the polymyxin-resistant 03-149.2 isolate by high-throughput sequencing and variant calling to *A. baumannii* ACICU

**Annotation by similarity to *A. baumannii* ACICU**	**Sequence**		**Effect**
	**03-149.1**	**03-149.2**	
Hypothetical Protein	CTTGAG	GCTTGAG	Frame shift
PmrB Sensor Kinase	AAGC	A	Amino acid deletion (A28-)
Phage-related protein	G	T	Amino acid substitution (I241L)

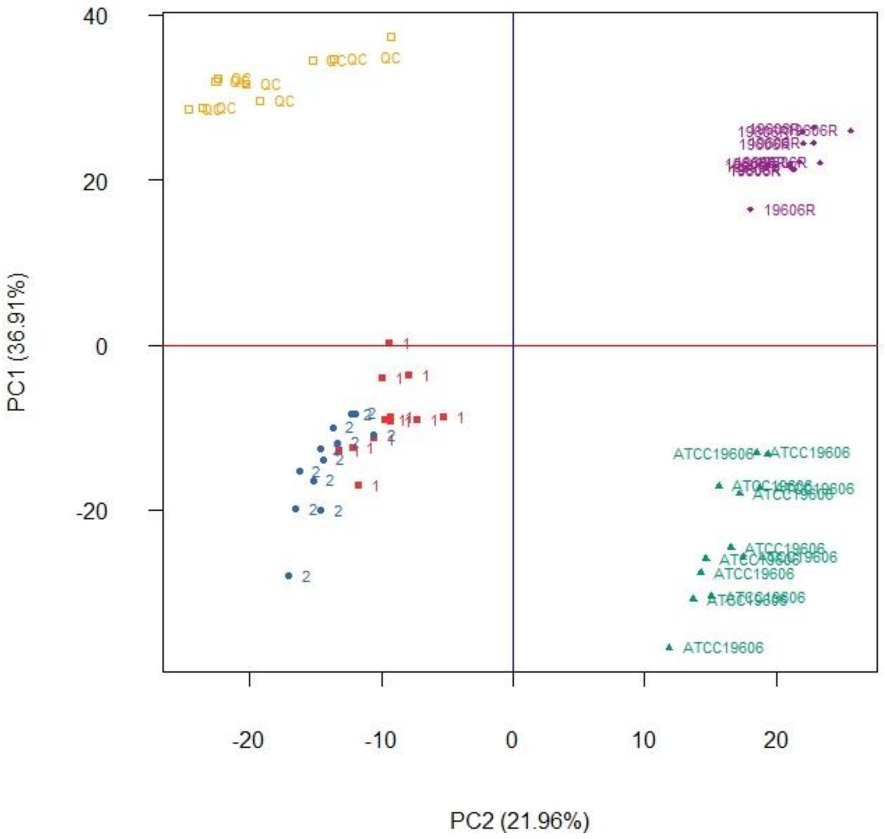

Supplementary Figure S1. PCA score plot of the untargeted metabolomics dataset of paired polymyxin-resistant 19606R and the wild-type ATCC 19606 and paired of polymyxin-resistant 03-149.2 (blue and labelled with "2") and polymyxin-susceptible 03-149.1 (red and labelled with "1") clinical isolates. Each data set for individual strains represents a total of 12 sample replicates (3 biological replicates and each with 4 technical replicates). Yellow, pool quality control samples; purple, polymyxin-resistant 19606R; green, ATCC 19606; blue, polymyxin-resistant 03-149.2; and red, polymyxin-susceptible 03-149.1.

(a)

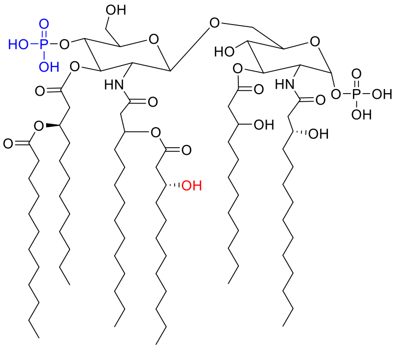

Exact Mass: 1729.12
-OH group: 1713.13
-H_2PO_3 group: 1649.15

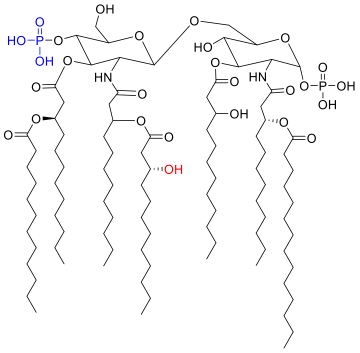

Exact Mass: 1883.26
-OH group: 1867.26
-H_2PO_3 group: 1803.29

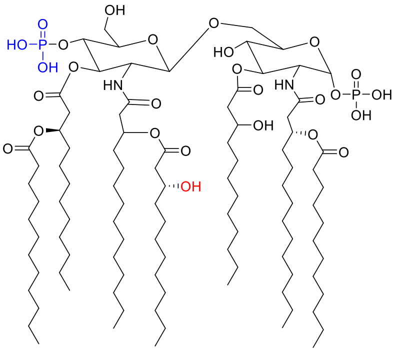

Exact Mass: 1911.29
-OH group: 1895.29
-H_2PO_3 group: 1831.32
+ Na: 1934.28

(b)

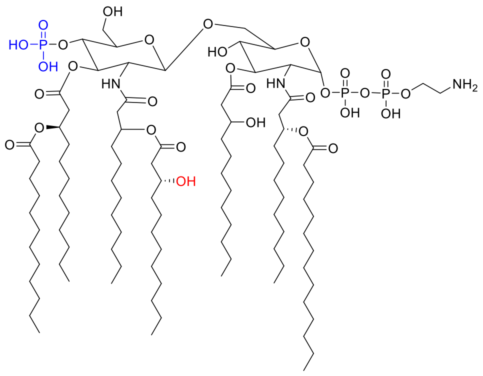

Exact Mass: 2006.26
-OH group: 1990.27

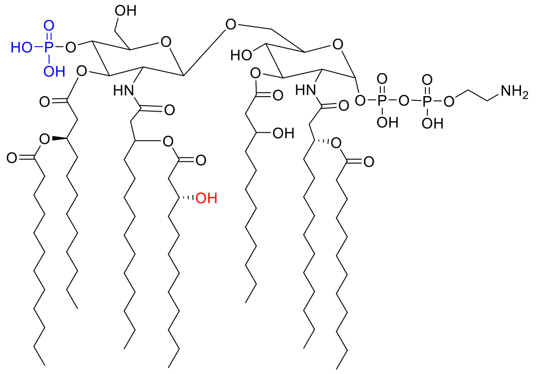

Exact Mass: 2034.30
-OH group: 2018.30
-H_2PO_3 group: 1954.33
+ Na: 2057.29

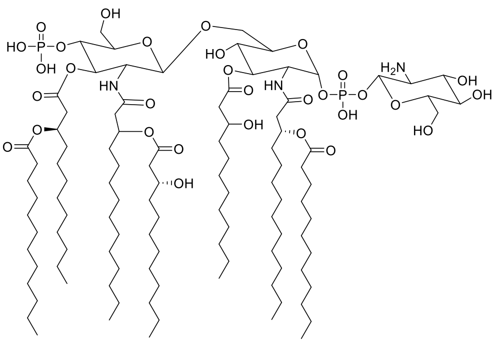

Exact Mass: 2072.36
+ Na: 2095.35

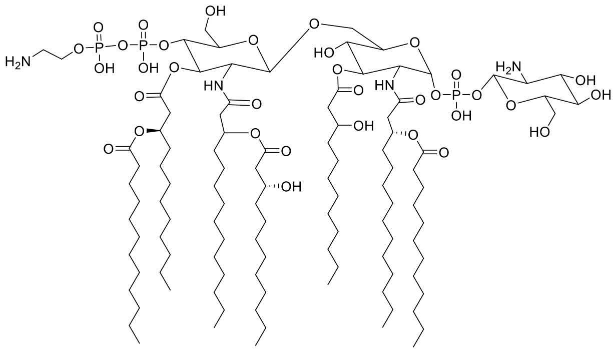

Exact Mass: 2195.36

Supplementary Figure S2. Structures of lipid A in clinical *A. baumannii* isolates (a) 03-149.1 and **(b)** 03-149.2.

References

1 Kim, O. S. *et al.* Introducing EzTaxon-e: a prokaryotic 16S rRNA gene sequence database with phylotypes that represent uncultured species. *Int. J. Syst. Evol. Micr.* **62**, 716-721 (2012).

2 Altschul, S. F. *et al.* Gapped BLAST and PSI-BLAST: a new generation of protein database search programs. *Nucleic Acids Res.* **25**, 3389-3402 (1997).
